# Editorial Note: Interferon regulatory factor 4 mediates nonenzymatic IRE1 dependency in multiple myeloma cells

**DOI:** 10.1371/journal.pbio.3003875

**Published:** 2026-08-03

**Authors:** The PLOS Biology Editors

After publication of this article [1], concerns were raised that the IRE1, XBP1s, and GAPDH panels in Fig 2D in [1] appear similar to the IRE1, XBP1s, and GAPDH panels in Fig 3H in [2] by the same author group.

The corresponding author confirmed that the IRE1, XBP1s, and GAPDH panels in Fig 2D in [1] are the same images as Fig 3H in [2], and that, as noted in the respective underlying blots in S1 Raw images in [1], the shIRE1 and shXBP1 lanes in these panels are also the same as in S3C Fig, and S4E Fig in [1]. They stated that the blots in [1] and [2] represent the same underlying samples and experimental conditions, and that these blots were obtained from a single collaborative experiment. They stated that the IRE1, XBP1s, and GAPDH blots served as shared control data, with the additional blot panels in [1] and [2] addressing different mechanistic questions through distinct proteins. The captions for Fig 2 and S3-S4 Figs in this article [1] are updated with this Editorial Note to reference that the Fig 2D, S3C Fig, and S4E Fig IRE1, XBP1s, and GAPDH panels are the same as in [2].

Upon review of the above information, the *PLOS Biology* Editors noted that not all of the blot panels presented in Fig 2D, S3C Fig and S4E Fig in [1] and Fig 3H in [2] that share a GAPDH loading control appear to be run on the same gel and blot. The corresponding author stated that the panels presented in these figures were obtained from different blots (3 blots in Fig 2D, 4 blots in S3C Fig, and 4 blots in S4E Fig) for which a single master set of samples was used, and they provided a schematic illustration of the blotting procedure (S1 File). These samples were normalized by buffer addition for total protein content as measured by BCA assay, simultaneously boiled in denaturing buffer, and loaded in equal volume onto several gels. Identical electrophoresis and electrotransfer conditions were used for all resulting gels and blots, which were also stained with Ponceau-S to visualize total transferred protein. They stated that the panels for IRE1, XBP1s, and GAPDH were derived from the same gel and corresponding blot. Furthermore, they clarified that the following figures also show proteins run on different blots, with the total number of different blots in each figure in parentheses: Fig 1F (2), Fig 1H (2), Fig 1J (2); Fig 2B (2); Fig 4D (5); Fig 5F (3); Fig 6G (3), Fig 6K (3); Fig S1B (2), Fig S1D (2), Fig S1F (2), Fig S1H (2); Fig S2B (2), Fig S2C, (2), Fig S2D (2); Fig S3F (2), Fig S3N (2), Fig S3O (3), Fig S3P (3); Fig S6D (2), Fig S6F (3), and Fig S6G (2).

In addition, there are errors in the Funding statement. The correct Funding statement is as follows: Genentech, Inc provided support for this study through salaries for all authors and funding for laboratory research. The funder had no additional role in study design, data collection and analysis, decision to publish, or preparation of the manuscript.

The *PLOS Biology* Editors issue this Editorial Note to inform readers of the above information.

**Fig 2:**
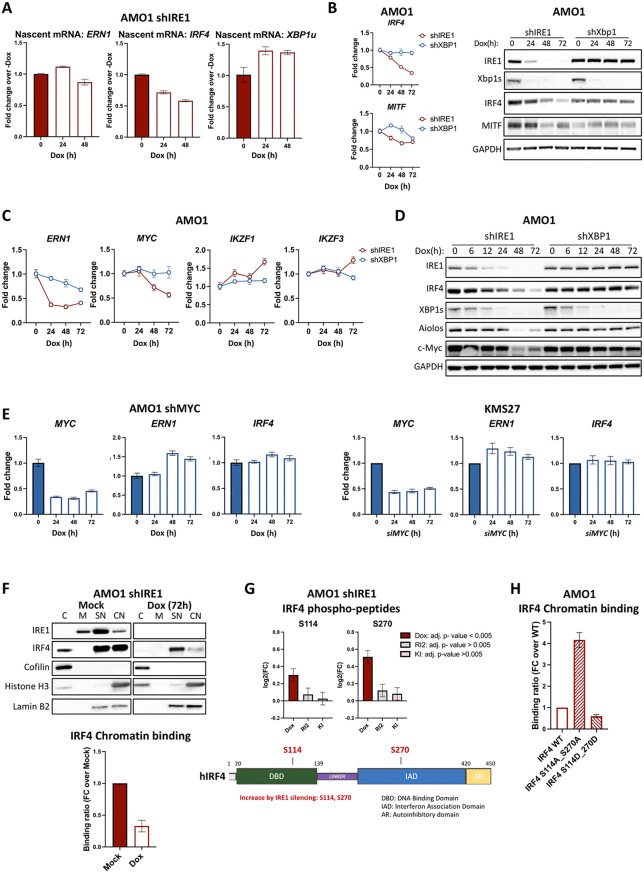
IRE1 silencing attenuates IRF4 activity. **(A)** Analysis of *IRF4* mRNA transcription by nuclear run-on. AMO1 shIRE1 Cl.1 cells were treated with 0.2 μg/mL Dox for the indicated time. Nascent RNA was labeled with a uridine analog, captured, and analyzed for *ERN1*, *IRF4,* and *XBP1*. Representative experiment out of three biological replicates. Data represented as mean ± SEM. **(B)** Effect of IRE1 and XBP1 silencing on MITF. AMO1 shIRE1 Cl.1 vs. shXBP1 Cl.1 cells were treated in a time-course of Dox (0.2 μg/mL) for up to 72 h and were analyzed by RT-qPCR for *MITF* and *IRF4* mRNA (left), and by IB for MITF protein levels (right). Data represented as mean ± SEM. **(C)** Effect of IRE1 and XBP1 silencing on Myc and Ikaros/Aiolos at the mRNA level. AMO1 shIRE1 Cl.1 and AMO1 shXBP1 Cl.1 cells were treated in the absence or presence of Dox (0.2 μg/mL) for the indicated time. Samples were analyzed by RT-qPCR for *MYC*, *IKZF1* (Ikaros transcript), and *IKZF3* (Aiolos transcript). Data represented as mean ± SEM. **(D)** Effect of IRE1 and XBP1 silencing on Myc protein levels. AMO1 shIRE1 Cl.1 and AMO1 shXBP1 Cl.1 cells were treated in the absence or presence of Dox (0.2 μg/mL) for the indicated time and were analyzed by IB for Myc and Aiolos. Ikaros could not be detected. The IRE1, XBP1s, and GAPDH panels were originally published in [2]. **(E)** Effect of MYC silencing on IRF4 in AMO1 and KMS27 cells. AMO1 cells were stably transfected with plasmids encoding Dox-inducible shRNA against Myc. AMO1 shMYC Cl. 7 cells (left) were treated with 0.2 μg/mL Dox for the indicated time. KMS27 cells (right) were nucleofected with or without siRNA against *MYC* for the indicated time. Cells were harvested and analyzed by RT-qPCR for *MYC*, *ERN1*, and *IRF4* mRNA. Data represented as mean ± SEM. **(F)** Effect of lRE1 silencing on IRF4 chromatin-binding. AMO1 shIRE1 Cl.1 cells were cultured in the absence or presence of 0.2 μg/mL Dox for 72 h. Cells were sequentially lysed into 4 subcellular fractions: C—cytoplasmic, M—Membrane, SN—Soluble Nuclear, CN—Chromatin-bound Nuclear. Nuclear fractions were analyzed by IB for IRE1 and IRF4 while Cofilin, Histone H3, and Lamin B2 served as fractionation controls. *Bottom:* The ratio of chromatin-bound over soluble nuclear IRF4 was determined by densitometry in 3 independent biological replicates and is depicted relative to mock-treated cells. **(G)** Nonenzymatic-IRE1-dependent phosphorylation sites on IRF4. AMO1 shIRE1 Cl.1 cells were treated in the absence or presence of 0.2 μg/mL Dox, 1 μM RI2 (a specific IRE1 RNase inhibitor), or 1 μM KI (a specific IRE1 kinase inhibitor) for 24 h and subjected to phosphoproteomic and global proteomics analyses. The phosphorylation data were normalized against the global proteomics data to control for changes in total protein levels. Four IRF4-phosphopeptides were identified, containing Ser114, Ser270, Ser241, Ser443, and Ser448. Only Ser114 and Ser270 sites were altered in a statistically significant fashion by IRE1 silencing. Top: Fold-change (log_2_FC) intensity of IRE1-dependent IRF4 phosphopeptides. Data represented as mean ± SE. Student *t* test: *p*-values were adjusted with the Benjamini–Hochberg method. Bottom: IRF4 protein schematic annotating IRE1-dependent phosphorylations. **(H)** Effect of substitution of S114 and S270 by alanine (phospho-deficient) or aspartic acid (phospho-mimetic) on IRF4’s chromatin-binding activity. AMO1 cells ectopically expressing Dox-inducible IRF4 WT, S114A_S270A, or S114D_S270D were incubated with 0.2 μg/mL Dox for 72 h, then fractionated and analyzed by IB (S2F Fig) as in Fig 2F. The ratio of chromatin-bound over soluble nuclear IRF4 was determined by densitometry in three independent biological replicates and is depicted relative to the WT IRF4 chromatin binding ratio. Data represented as mean ± SEM. Data underlying this figure can be found in S1 Data, S1 Raw images, and MassIVE repository (MSV000095907, https://doi.org/10.25345/C5WH2DS2R).

## Supporting information

S3 Fig IRF4 silencing recapitulates the anti-proliferative effect of IRE1 knockdown. (A) Validation of IRF4 depletion by IRF4 silencing. AMO1 shIRE1 Cl.1 or shIRF4 Cl.1 cells were stably transfected with plasmids encoding Dox-inducible shRNAs against either IRF4 or IRE1. The cells were treated with Dox (0.2 μg/mL) for the indicated time. Samples were analyzed by IB for IRE1 and IRF4. **(B)** Effect of IRF4, IRE1 or NTC silencing on in vitro spheroid growth of KMS27 and validation of IRF4 silencing. KMS27 cells were stably transfected with plasmids encoding Dox-inducible shRNAs against either IRF4 (purple) or non-targeting control (blue). *Left, middle:* KMS27 shNTC, shIRE1, or shIRF4 cells were treated with Dox (0.2 μg/mL) for the indicated time. Samples were analyzed by RT-qPCR and IB for IRE1/*ERN1* and IRF4. *Right:* Growth of these cells in the absence (closed symbols) or presence (open symbols) of Dox (0.2 μg/mL) was compared to that of cells expressing shRNAs against IRE1 or NTC. Spheroid growth, depicted as FC confluence, was monitored by time-lapse microscopy in an IncuCyte instrument and values represent mean ± SEM. **(C)** Cell death markers. AMO1 shIRE1 Cl.1, shIRF4 Cl.1, or shXBP1 Cl.1 cells were treated in the absence or presence of Dox (0.2 μg/mL) for up to 72 h and post-nuclear lysates were analyzed by IB for Lamin A/C and PARP1 cleavage as well as Histones. GAPDH is used as a loading control. The IRE1, XBP1s, and GAPDH panels were originally published in [2]. **(D)** Effect of IRE1 or IRF4 silencing on caspase activation. AMO1 shIRE1 Cl.1 or shIRF4 Cl.1 cells treated in the absence (filled bars) or presence of Dox (0.2 μg/mL) for 72 h were analyzed for caspase activity by Caspase-Glo assays. Representative replicate. Values presented as mean ±SEM. **(E)** Effect of IRE1 or IRF4 silencing on Annexin V/ PI staining. AMO1 shIRE1 Cl.1 or shIRF4 Cl.1 cells were treated with Dox (0.2 μg/mL) for the indicated times. Cells were then stained with FITC-Annexin V and PI and analyzed by flow cytometry for early apoptotic (FITC+ PI-), late apoptotic (FITC+ PI+), and necrotic (FITC- PI+) cells. **(F)** Q-VD blockade of caspase cleavage during IRE1 or IRF4 silencing. AMO1 shIRE1 Cl.1 or shIRF4 Cl.1 cells were treated with Dox (0.2 μg/mL) for 72 h in the absence or presence of the pan-caspase inhibitor Q-VD (50 μM). Samples were analyzed by IB for caspase and PARP1 cleavage. **(G)** Viability of IRE1 or IRF4-deficient cells during Q-VD treatment. Cells treated as in S3F Fig were analyzed for plasma membrane integrity/viability by trypan-blue staining and quantified in a Vi-Cell machine. **(H)** In vitro spheroid growth of IRF4 or IRE1 deficient cells during Q-VD treatment. AMO1 shIRE1 Cl.1 or shIRF4 Cl.1 cells were cultured in the absence (filled symbols) or presence (open symbols) of Dox (0.2 μg/mL) with or without Q-VD (50 μM) and spheroid cell growth, depicted as FC confluence, was monitored by time-lapse microscopy in an IncuCyte instrument. Values presented as mean ±SEM. **(I)** Validation of caspase inactivation. Samples from S3H Fig were analyzed for caspase activity by Caspase 3/7-Glo assay. Values presented as mean ±SEM. **(J)** Effect of IRE1 and IRF4 silencing on cell divisions in KMS27 cells. KMS27 shIRE1, shIRF4, or shNTC were stained with a CFSE-type dye and incubated in the absence (filled curves) or presence (open curves) of Dox (0.2 μg/mL) and analyzed by flow cytometry. Etoposide (Eto, 25 μM, dashed line) was used as a non-proliferative control. Representative experiment. **(K)** Control panels for Fig 3C. Quantification of apoptotic (left) versus dividing (right) cells. **(L)** Cell cycle profiling in KMS27 cells. KMS27 shIRF4 cells were incubated in the absence (filled symbols) or presence (open symbols) of Dox (0.2 μg/mL) for the indicated times, EtOH fixated and PI stained before analyzed by flow cytometry. The indicated cell cycle phases were determined according to univariate (DNA content) modeling. **(M)** Effect of Myc silencing on in vitro spheroid growth and cell cycle in AMO1 cells. Left: Growth of AMO1 shMYC Cl. 7 (yellow) in the absence of any treatment (closed symbols) was compared to that of cells treated with Dox (0.2 μg/mL) as well as to the growth of AMO1 shIRE1, shIRF4, and shNTC cells in the presence of Dox. Spheroid growth, depicted as FC confluence, was monitored by time-lapse microscopy in an IncuCyte instrument and values represent mean ± SEM. *Right:* G1-phase profiling of the same cell lines. Cells were incubated in the presence of absence of Dox for 48 h, EtOH fixated and PI stained before analyzed by flow cytometry. G1 cell cycle phase was determined according to univariate (DNA content) modeling. Values represented as mean ± SEM. **(N)** CDK2 phosphorylations during IRE1 or IRF4 silencing in KMS27 cells. KMS27 shIRE1 Cl.9, shIRF4 Cl.20, or shXBP1 Cl.22 cells were treated in the presence or absence of Dox (0.2 μg/mL) in a time course of 0, 6, 24, 48, and 72 h and analyzed by IB for total CDK2 and pT160 CDK2. **(O)** IRF4 silencing effect on inhibitory and activating CDK phosphorylations. AMO1 shIRF4 Cl.1 cells were cultured in the absence (filled symbols) or presence (open symbols) of Dox (0.2 μg/mL) and collected at the indicated time post-treatment to be analyzed by IB for total CDK6, pT24 CDK6 (inhibitory), total CDK2, pT14 CDK2 (inhibitory), and pT160 CDK2 (activating). Bottom panels: quantification by densitometry. **(P)** Cdc25A protein expression during IRF4 silencing. AMO1 shIRF4 Cl.1 or shNTC cells were treated in the absence or presence of Dox (0.2 μg/mL) for the indicated time. Cells were analyzed by IB for total or phosphoT124-Cdc25A as well as total CDK levels. Right: quantification of Cdc25A by densitometry in shNTC (filled symbols) or shIRF4 (open symbols) cells. The data underlying this figure can be found in S1 Data**, Zenodo (**https://doi.org/10.5281/zenodo.14928364**),** and S1 Raw images.
(TIFF)

S4 Fig IRE1 and IRF4 regulate a highly overlapping set of cell cycle genes. (A) Validation of samples from Fig 4. AMO1 shIRF4 Cl.1 or shNTC bulk RNA-seq analysis (addressed in Fig 4), validates that IRF4 transcripts were depleted during the time course of silencing, as well as transcripts of known IRF4 targets. i.e., *MYC*, *PRDM1* (transcript of BLIMP1) and *XBP1*. **(B)** Complete IRF4 GSEA (Hallmark). GSEA analysis was performed as described in Fig 4A. Shown all enriched datasets. In gray, gene sets with FDR > 0.02. **(C)** ORA of AMO1 shIRE1 (top) and shIRF4 (bottom). Overrepresentation analysis of the transcriptomics results was performed using the Interactive analysis enrichment tool. Depicted are select GO terms. **(D)** Overlap of genes between IRE1 and IRF4 Knockdowns. After GSEA in Fig 4A, Leading Edge Genes (S2 Table), representing those contributing most significantly to the enrichment of the given gene sets, were extracted for both genetic backgrounds. Shown: Venn diagrams illustrating the intersection between the Leading-Edge Genes for “G1S Cell Cycle Control” (Wiki Pathways) and “Unfolded Protein Response” (UPR; Hallmark) between IRE1 knockdown (shIRE1) and IRF4 knockdown (shIRF4) backgrounds. Middle: Venn diagram representing all DNA repair GO term genes identified in the two backgrounds and their overlap. **(E)** UPR downregulation was validated by IB in both genetic backgrounds. Samples treated as in Fig 4D were analyzed by IB for pIRE1 as well as PERK and ATF6 pathway proteins. The IRE1, XBP1s, and GAPDH panels were originally published in [2]. **(F)** Effect of IRE1 or IRF4 knockdown on mRNA expression of DNA repair genes. Heatmap depicting the top 100 downregulated genes match to “DNA repair” GO term in the transcriptomics analyses described in Fig 4A. **(G)** Complete IRF4 GSEA (Hallmark) analysis in KMS27 cells. Analysis was performed as described in Fig 4A for KMS27 cells. Shown all enriched datasets. In gray, gene sets with FDR > 0.02. *Right:* Overlap of Leading-Edge Genes from GSEA analyses in IRF4-deficient AMO1 and KMS27 cells. After GSEA in Fig 4A, Leading Edge Genes (S2 Table), representing those contributing most significantly to the enrichment of the given gene sets, were extracted for both cell lines. Shown: Venn diagrams illustrating the intersection between the Leading-Edge Genes for Hallmark “E2F-Targets” and “G2/M Checkpoint” between KMS27 and AMO1 cells deficient in IRF4. The data underlying this figure can be found in S1 Data, S1 Raw images, and GEO repository (GSE288674).
(TIFF)

S1 File Immunoblotting layout. This diagram was also published in [3].
(PDF)
