# Supplementary figures and images for: Editorial Note: Interferon regulatory factor 4 mediates nonenzymatic IRE1 dependency in multiple myeloma cells
Source: PLoS Biol. 2026 Aug 3;24(8):e3003875. doi: 10.1371/journal.pbio.3003875 (PMC13432093; doi:10.1371/journal.pbio.3003875)

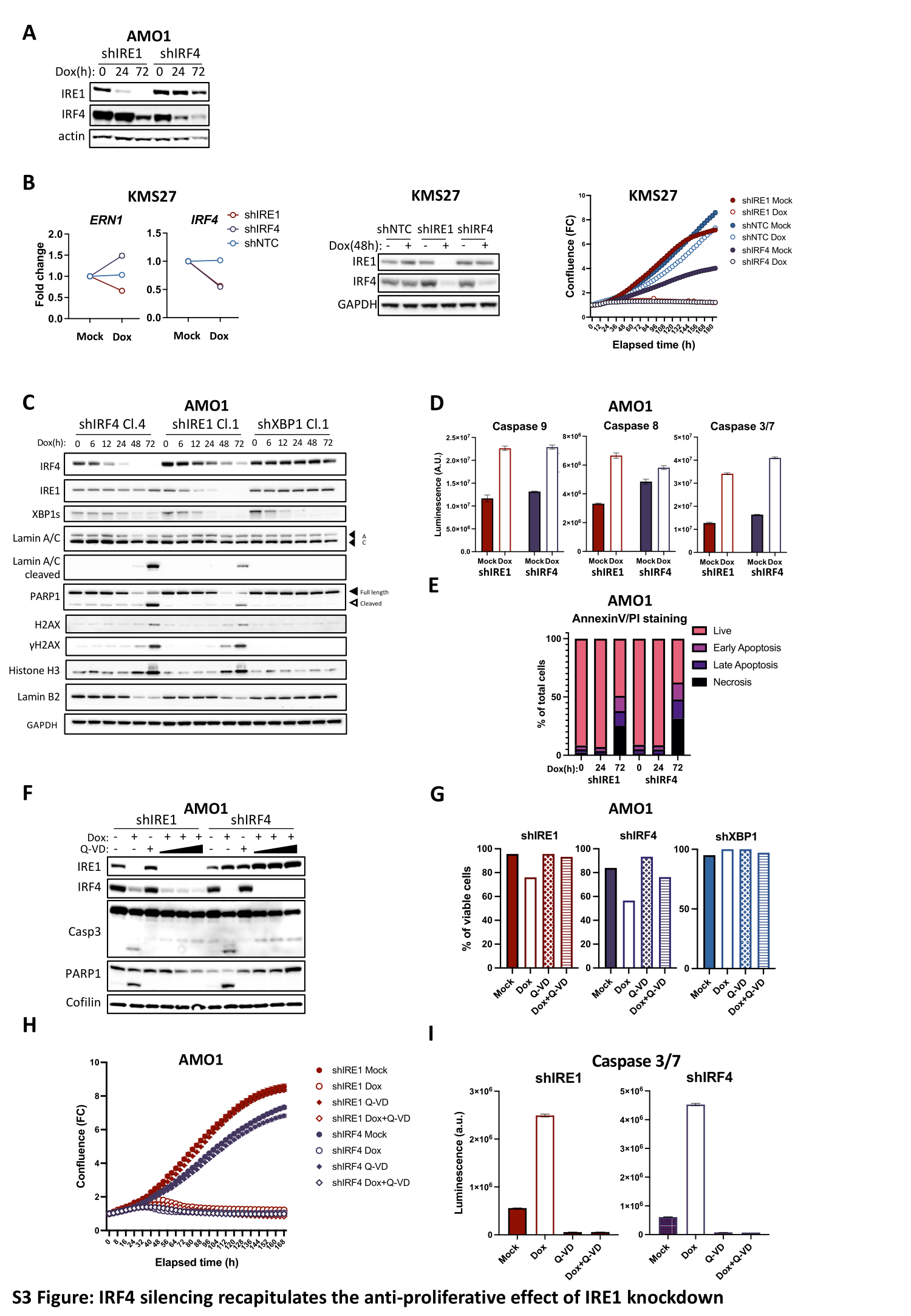

Supplement: S3 Fig — (A) Validation of IRF4 depletion by IRF4 silencing. AMO1 shIRE1 Cl.1 or shIRF4 Cl.1 cells were stably transfected with plasmids encoding Dox-inducible shRNAs against either IRF4 or IRE1. The cells were treated with Dox (0.2 μg/mL) for the indicated time. Samples were analyzed by IB for IRE1 and IRF4. (B) Effect of IRF4, IRE1 or NTC silencing on in vitro spheroid growth of KMS27 and validation of IRF4 silencing. KMS27 cells were stably transfected with plasmids encoding Dox-inducible shRNAs against either IRF4 (purple) or non-targeting control (blue). Left, middle: KMS27 shNTC, shIRE1, or shIRF4 cells were treated with Dox (0.2 μg/mL) for the indicated time. Samples were analyzed by RT-qPCR and IB for IRE1/ERN1 and IRF4. Right: Growth of these cells in the absence (closed symbols) or presence (open symbols) of Dox (0.2 μg/mL) was compared to that of cells expressing shRNAs against IRE1 or NTC. Spheroid growth, depicted as FC confluence, was monitored by time-lapse microscopy in an IncuCyte instrument and values represent mean ± SEM. (C) Cell death markers. AMO1 shIRE1 Cl.1, shIRF4 Cl.1, or shXBP1 Cl.1 cells were treated in the absence or presence of Dox (0.2 μg/mL) for up to 72 h and post-nuclear lysates were analyzed by IB for Lamin A/C and PARP1 cleavage as well as Histones. GAPDH is used as a loading control. The IRE1, XBP1s, and GAPDH panels were originally published in [2]. (D) Effect of IRE1 or IRF4 silencing on caspase activation. AMO1 shIRE1 Cl.1 or shIRF4 Cl.1 cells treated in the absence (filled bars) or presence of Dox (0.2 μg/mL) for 72 h were analyzed for caspase activity by Caspase-Glo assays. Representative replicate. Values presented as mean ±SEM. (E) Effect of IRE1 or IRF4 silencing on Annexin V/ PI staining. AMO1 shIRE1 Cl.1 or shIRF4 Cl.1 cells were treated with Dox (0.2 μg/mL) for the indicated times. Cells were then stained with FITC-Annexin V and PI and analyzed by flow cytometry for early apoptotic (FITC+ PI-), late apoptotic (FITC+ PI+), and n [file pbio.3003875.s001.tiff]

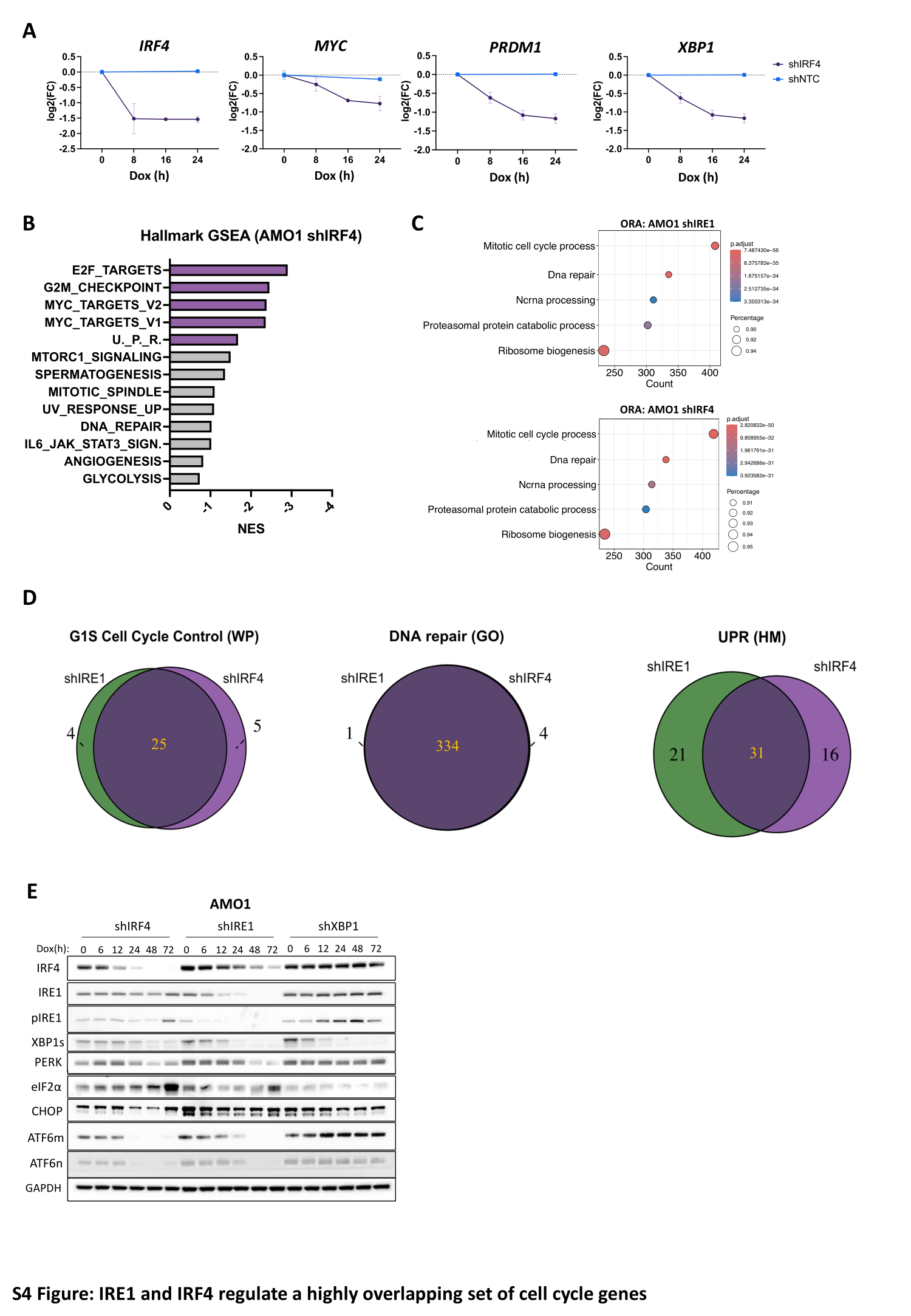

Supplement: S4 Fig — (A) Validation of samples from Fig 4. AMO1 shIRF4 Cl.1 or shNTC bulk RNA-seq analysis (addressed in Fig 4), validates that IRF4 transcripts were depleted during the time course of silencing, as well as transcripts of known IRF4 targets. i.e., MYC, PRDM1 (transcript of BLIMP1) and XBP1. (B) Complete IRF4 GSEA (Hallmark). GSEA analysis was performed as described in Fig 4A. Shown all enriched datasets. In gray, gene sets with FDR > 0.02. (C) ORA of AMO1 shIRE1 (top) and shIRF4 (bottom). Overrepresentation analysis of the transcriptomics results was performed using the Interactive analysis enrichment tool. Depicted are select GO terms. (D) Overlap of genes between IRE1 and IRF4 Knockdowns. After GSEA in Fig 4A, Leading Edge Genes (S2 Table), representing those contributing most significantly to the enrichment of the given gene sets, were extracted for both genetic backgrounds. Shown: Venn diagrams illustrating the intersection between the Leading-Edge Genes for “G1S Cell Cycle Control” (Wiki Pathways) and “Unfolded Protein Response” (UPR; Hallmark) between IRE1 knockdown (shIRE1) and IRF4 knockdown (shIRF4) backgrounds. Middle: Venn diagram representing all DNA repair GO term genes identified in the two backgrounds and their overlap. (E) UPR downregulation was validated by IB in both genetic backgrounds. Samples treated as in Fig 4D were analyzed by IB for pIRE1 as well as PERK and ATF6 pathway proteins. The IRE1, XBP1s, and GAPDH panels were originally published in [2]. (F) Effect of IRE1 or IRF4 knockdown on mRNA expression of DNA repair genes. Heatmap depicting the top 100 downregulated genes match to “DNA repair” GO term in the transcriptomics analyses described in Fig 4A. (G) Complete IRF4 GSEA (Hallmark) analysis in KMS27 cells. Analysis was performed as described in Fig 4A for KMS27 cells. Shown all enriched datasets. In gray, gene sets with FDR > 0.02. Right: Overlap of Leading-Edge Genes from GSEA analyses in IRF4-deficient AMO1 and KMS27 cells. After GSEA in F [file pbio.3003875.s002.tiff]
